# Sex-Specific Molecular and Genomic Responses to Endocrine Disruptors in Aquatic Species: The Central Role of Vitellogenin

**DOI:** 10.3390/genes16111317

**Published:** 2025-11-02

**Authors:** Faustina Barbara Cannea, Cristina Porcu, Maria Cristina Follesa, Alessandra Padiglia

**Affiliations:** 1 Department of Life and Environmental Sciences (DiSVA), Biomedical Section, University of Cagliari, Cittadella Universitaria di Monserrato, Monserrato, 09042 Cagliari, Italy; faustinab.cannea@unica.it; 2 Department of Life and Environmental Sciences (DiSVA), Marine Bioecology Section, University of Cagliari, Via T. Fiorelli 1, 09126 Cagliari, Italy; cristina.porcu@unica.it (C.P.); follesac@unica.it (M.C.F.)

**Keywords:** endocrine-disrupting chemicals, vitellogenin, biomarkers, sex-specific gene expression, transcriptomics, epigenetic regulation, omics-based integration, ecogenomics, adverse outcome pathway, aquatic organisms

## Abstract

Endocrine-disrupting chemicals (EDCs) are widespread contaminants that interfere with hormonal signaling and compromise reproductive success in aquatic organisms. Vitellogenin (VTG) is one of the most widely established biomarkers of estrogenic exposure, especially in males and juveniles. However, evidence from multi-omics studies indicates that VTG induction occurs within broader transcriptional and regulatory networks, involving genes such as *cyp19a1* (aromatase), *cyp1a* (cytochrome P4501A), and other stress-responsive genes, underscoring the complexity of endocrine disruption. This review focuses on nuclear receptor isoforms, including estrogen receptor alpha (ERα), estrogen receptor beta (ERβ), and androgen receptor (AR) variants. We examine the diversification of *vtg* gene repertoires across teleost genomes and epigenetic mechanisms, such as DNA methylation and microRNAs, that modulate sex-dependent sensitivity. In addition, we discuss integrative approaches that combine VTG with transcriptomic, epigenetic, and histological endpoints. Within the Adverse Outcome Pathway (AOP) and weight-of-evidence (WoE) frameworks, these strategies provide mechanistic links between receptor activation and reproductive impairment. Finally, we outline future directions, focusing on the development of sex-specific biomarker panels, the integration of omics-based data with machine learning, and advances in ecogenomics. Embedding molecular responses into ecological and regulatory contexts will help bridge mechanistic insights with environmental relevance and support sustainability goals such as SDG 14 (Life Below Water).

## 1. Introduction

Aquatic ecosystems are increasingly threatened by EDCs, a diverse group of contaminants that interfere with hormonal signaling and homeostasis in wildlife [1,2]. Their presence in surface waters and sediments has been widely documented, together with their capacity to compromise reproduction, growth, and development in aquatic organisms [3,4,5].

An essential dimension of endocrine disruption research is the consideration of sex as a biological variable. Males and females differ in circulating hormone levels, reproductive physiology, receptor distribution, metabolic capacity, and epigenetic regulation [6,7,8]. These differences shape sex-dependent molecular responses, whereby EDCs trigger divergent transcriptional, epigenetic, and physiological effects [9,10]. This variability has profound implications for reproductive fitness and population resilience, underscoring the need to incorporate sex-based perspectives into ecotoxicological assessment [11,12].

Among molecular biomarkers, the yolk precursor protein VTG is the most established indicator of estrogenic activity [13,14]. Synthesized in the liver of oviparous females under natural conditions, VTG is normally absent in males and juveniles but becomes strongly induced by exposure to estrogenic EDCs [11,12]. This makes VTG a robust and sensitive biomarker, extensively applied in ecotoxicology through both protein-based assays (ELISA, Western blotting) and transcript-level analyses (qPCR, RNA-Seq) [15,16,17,18]. Nevertheless, interpretation requires careful consideration of sex, reproductive stage, species variability, and environmental context.

Although fish dominate the literature, evidence from other taxa, such as mollusks, crustaceans, and amphibians, confirms that endocrine disruption is a cross-phyla phenomenon [19,20,21,22,23]. These findings emphasize the need for biomarker strategies that extend beyond fish and incorporate alternative molecular endpoints. Recent advances in omics technologies reveal that EDC responses extend beyond VTG and involve nuclear receptors, steroidogenic enzymes, detoxification pathways, and stress-related genes [24,25,26]. Placing VTG within this broader multi-omics framework underscores its central role and highlights the need for integrative approaches to capture the complexity of endocrine disruption.

The aim of this review is to provide an integrated perspective on sex-specific molecular responses to EDCs in aquatic organisms, with particular emphasis on the ecotoxicological relevance of VTG and its integration into multi-omics approaches. Ultimately, this work seeks to advance ecotoxicological assessment and contribute to global sustainability efforts, particularly the United Nations Sustainable Development Goal (SDG) 14: Life Below Water [27].

### Methodology

This review was conducted as a narrative synthesis of the scientific literature. Relevant studies were retrieved from PubMed, Scopus, and Web of Science using combinations of the following keywords: “endocrine-disrupting chemicals” OR “EDCs”, “sex-specific responses”, “vitellogenin” OR “VTG”, “multi-omics”, “fish”, “mollusks”, “crustaceans”, and “aquatic organisms”. The main search covered the period from 2000 to 2025 to capture both seminal and most recent contributions. However, earlier key studies of fundamental relevance (e.g., discovery of VTG as a biomarker, initial reports of EDC effects) were also included to provide historical and conceptual context. In total, approximately 1200 records were initially retrieved, 300 screened in detail, and 189 included in this review.

## 2. Endocrine Disruptors in Aquatic Environments

EDCs are recognized as priority pollutants due to their ability to interfere with hormonal signaling and reproduction in aquatic organisms [28,29]. Although fish are particularly sensitive because of their constant waterborne exposure, invertebrates such as bivalves and crustaceans also display endocrine-sensitive processes, including vitellogenin (VTG)-like protein synthesis and disrupted steroidogenic pathways [30]. EDCs originate from agriculture, industry, urbanization, and wastewater discharge, with most ecotoxicological research focusing on four main categories: pesticides, plasticizers, pharmaceuticals, and industrial contaminants.

### 2.1. Main Sources of EDCs

Representative examples illustrate the breadth of EDC impacts. Legacy pesticides such as dichlorodiphenyltrichloroethane (DDT) persist in sediments and retain estrogenic activity decades after their ban [31]. Atrazine, one of the most widely used herbicides, alters aromatase activity and disrupts sex steroid balance, leading to feminization in fish and amphibians [32,33].

Plasticizers such as bisphenol A (BPA) and nonylphenol bind estrogen receptors and induce VTG in male fish, with field studies linking nonylphenol exposure to intersex conditions in wild roach (*Rutilus rutilus*) populations downstream of wastewater treatment plants [34,35]. In mollusks, BPA disrupts embryonic development in *Mytilus galloprovincialis* and alters shell formation and serotonergic signaling [36], whereas induction of VTG-like proteins remains inconsistent, with some studies reporting biomarker potential and others finding no induction even under potent EE2 exposure [37].

Among synthetic estrogens, 17α-ethinylestradiol (EE2) is particularly concerning, being resistant to degradation and active at ng/L concentrations. A landmark whole-lake experiment in Canada demonstrated that chronic EE2 exposure caused the collapse of *Pimephales promelas* populations, with recovery only after exposure ceased [38,39]. More broadly, reviews emphasize the profound impacts of estrogenic EDCs on reproduction in wildlife and humans [40].

Industrial contaminants such as polychlorinated biphenyls (PCBs) and dioxins act mainly through activation of the aryl hydrocarbon receptor (AhR), disrupting reproduction and development [41], while heavy metals such as cadmium and mercury impair steroid biosynthesis in both fish and mollusks.

Per- and polyfluoroalkyl substances (PFAS) are another class of persistent contaminants that interfere with thyroid and reproductive hormone regulation in fish [42,43] and in mollusks, where experimental exposures demonstrated immune and endocrine-related effects [44,45].

### 2.2. Exposure Pathways in Aquatic Organisms

EDCs enter aquatic organisms through multiple routes, including gill diffusion, dermal absorption, and oral ingestion. Transporters such as solute carriers (SLCs) and ATP-binding cassette (ABC) efflux pumps regulate bioavailability, while the intestinal microbiota can regenerate active estrogens from conjugated forms [46,47,48,49]. Lipophilic compounds (e.g., PCBs, organochlorine pesticides) accumulate in liver and gonads, enabling maternal transfer via VTG [50,51]. In contrast, polar contaminants such as PFAS circulate bound to plasma proteins and persist through enterohepatic recirculation [48,52,53]. In invertebrates, the hepatopancreas serves as the main detoxification organ, expressing Phase I/II enzymes and ABC transporters [54,55,56,57,58]. Crustaceans accumulate contaminants via gills and diet, with subsequent disruption of the ecdysteroid–methyl farnesoate axis [59,60,61]. Bioaccumulation and trophic transfer enhance contaminant transfer, with bivalves and crustaceans acting both as sentinels and vectors within marine food webs [62,63].

### 2.3. Physiological and Reproductive Effects of EDCs

At the molecular level, EDCs act through multiple mechanisms, including receptor binding, interference with steroidogenic enzymes, and epigenetic modifications [64,65,66,67,68,69,70,71,72,73,74]. These perturbations cascade into intersex gonads, impaired spermatogenesis with reduced sperm motility, abnormal oogenesis, altered sex steroid ratios, secondary sexual trait modifications, and reduced fecundity, with well-documented cases in fish populations exposed to estrogenic effluents and EE2. Long-term surveys in UK rivers have linked the feminization of wild *R. rutilus* populations directly to wastewater effluents, providing evidence that spans from ER activation to *vtg* gene induction and histopathological alterations. Similarly, the whole-lake EE2 experiment in Canada showed that molecular and histological perturbations scaled up to a demographic collapse, with recovery occurring only after exposure ceased [38,75].

In addition to freshwater fish, evidence from marine species highlights comparable effects. In Atlantic cod (*Gadus morhua*), multi-omics analyses revealed hepatic reprogramming of lipid metabolism and immunity following EE2 exposure [76]. European flounder (*Platichthys flesus*) collected from polluted estuaries exhibited co-induction of VTG, choriogenins, and heat shock proteins, confirming field-level estrogenic impacts [77]. These findings underscore that endocrine disruption in marine fish parallels observations in freshwater taxa, with similar molecular-to-population cascades.

In bivalves, exposure to estrogenic and anti-androgenic compounds alters gametogenesis, steroid metabolism, and larval development. Transcriptomic studies in *Mytilus edulis* and *M. galloprovincialis* revealed perturbations in lipid metabolism, serotonin/prostaglandin signaling, and vitellogenesis [19,78]. However, induction of VTG-like proteins remains inconsistent across species, underscoring the limitations of VTG as a universal biomarker in invertebrates [19].

In crustaceans, pesticides and organotins disrupt reproduction by interfering with the ecdysteroid–methyl farnesoate (MF) axis through the EcR/RXR receptor complex. These effects manifest as impaired molting, abnormal vitellogenesis, and reduced fertility, as supported by evidence on vitellogenin-like proteins in invertebrates [79], reviews of endocrine disruption in crustaceans [80], updated studies on organotin toxicity [81], and mechanistic insights into endocrine regulation of reproduction [82]. EcR/RXR-mediated signaling is a known target of endocrine toxicants [83], and experimental exposure to methyl farnesoate (MF) and 20-hydroxyecdysone (20E) in *Marsupenaeus japonicus* larvae has further confirmed the vulnerability of these pathways to disruption [84]. Moreover, crustacean endocrine physiology differs substantially from vertebrates, requiring taxon-specific evaluation of biomarkers [85].

Overall, these findings highlight the need for integrative biomarker approaches combining VTG, steroidogenic enzymes, receptor transcripts, and epigenetic endpoints. Within the Adverse Outcome Pathway (AOP) framework, such biomarkers can be linked to higher-level outcomes, thereby supporting regulatory risk assessment [86,87]. Collectively, they provide a comparative perspective across phyla, showing how endocrine-disrupting chemicals impact aquatic organisms at multiple levels of biological organization (Table 1).

## 3. Sex-Specific Molecular Responses to EDCs

The molecular effects of EDCs are strongly sex-dependent, reflecting dimorphisms in endocrine physiology, receptor isoform composition, gene repertoires, and epigenetic control. These differences lead to divergent transcriptional, epigenetic, and metabolic responses in males and females exposed to the same contaminant. Understanding such pathways is essential for interpreting biomarkers, refining ecotoxicological assessments, and developing predictive strategies aligned with genomics-based strategies.

### 3.1. Nuclear Receptor Isoforms (ERα, ERβ, and AR Variants)

Estrogen and androgen receptors are primary mediators of endocrine disruption, and their isoform diversity underpins sex-specific responses. In teleosts, gene duplication produced multiple ER isoforms, including ERα (*esr1*) and two ERβ paralogs (*esr2a*, *esr2b*), which differ in tissue distribution and transcriptional roles [88,89]. Functional studies confirm that these isoforms are not redundant, as each fulfills distinct roles in reproduction and development.

In zebrafish *(Danio rerio*), *esr1* knockout abolishes VTG synthesis and impairs ovarian maturation, whereas disruption of *esr2a/b* affects fertility, sexual differentiation, and gonadal morphology [90]. ERα is therefore the main driver of hepatic *vtg* induction, while ERβ isoforms modulate reproduction and development. ER isoforms also differ in ligand-binding affinities: ERα has high affinity for natural estrogens such as 17β-estradiol (E2), whereas ERβ paralogs often respond more strongly to synthetic estrogens such as EE2 or BPA [91]. In addition to nuclear ERs, membrane-associated receptors also play a role, with GPER (*gper1*) mediating rapid signaling and inhibiting oocyte maturation in *D. rerio* [92]. Androgen receptors have also diversified, with ARα and ARβ encoded by distinct genes. ARα is predominant in gonadal development, whereas ARβ contributes mainly to neural and behavioral traits [92].

Anti-androgenic contaminants strongly affect AR-regulated pathways: in stickleback (*Gasterosteus aculeatus*), suppression of the AR-dependent protein spiggin by flutamide or fungicides directly impairs male fertility [93]. Other receptors also participate in sex-specific responses. PGRMC1 and mPRα are required for normal oocyte maturation in *D. rerio* [94,95], while the glucocorticoid receptor responds to salinity and immune stressors in several teleosts, including large yellow croaker (*Larimichthys crocea*) [96]. Cross-talk further modulates effects. Activation of AhR by dioxin-like PCBs antagonizes ER-mediated transcription and reduces *vtg* induction in males [97,98].

Emerging contaminants such as PFAS also interact with these receptors, raising concern for their disruptive potential across taxa [99]. In invertebrates, BPA and nonylphenol induce *vtg*-like and ER-related transcripts in *M. galloprovincialis*, while in crustaceans organotins disrupt nuclear receptor-mediated control of vitellogenesis and molting [61,71].

### 3.2. Diversification of vtg Gene Repertoires in Teleosts

The *vtg* gene family exemplifies how genomic architecture shapes biomarker responses. Successive genome duplications in teleosts generated multiple paralogs with distinct regulatory profiles. In *D. rerio*, eight *vtg* genes are organized into three types: five type I (*vtg1*, *4*–*7*), two type II (*vtg2*, *8*), and one type III (*vtg3*) [100,101]. It should be noted that two different classification systems are used in the literature: the designation of type I–III VTGs refers to structural features and domain organization, whereas the clade nomenclature (Aa, Ab, C) reflects phylogenetic relationships among teleost paralogs. Functional validation using CRISPR–Cas9 demonstrated that both type I and type III paralogs play essential roles in reproduction and early development, with *vtg1* and *vtg3* knockouts severely impairing fertility, embryo survival, and gonadal differentiation [102]. In salmonids, which underwent an additional genome duplication, large *vtg* multigene arrays have been described, often organized in chromosomal clusters [103]. Some paralogs are highly estrogen-responsive, while others show minimal induction, consistent with subfunctionalization [104,105]. It has been proposed that certain paralogs may function as molecular buffers, supporting reproduction under chronic contaminant exposure but reducing plasticity in fluctuating environments [105]. This genomic diversification helps explain interspecific variation in VTG responses: for instance, *D. rerio* and medaka (*Oryzias latipes*) respond to ng/L estrogens, whereas salmonids typically require higher exposures [106]. From this perspective, genomic architecture provides a conceptual basis for interpreting interspecific differences in VTG induction and ecotoxicological sensitivity. Recent genomic analyses in Sichuan bream (*Sinibrama taeniatus*) identified six *vtg* genes, classified into types I–III based on subdomain structure, and confirmed that promoter sequences contain multiple estrogen response elements (EREs). The abundance of EREs correlated with the responsiveness of *vtg* expression to estrogen, with ERα emerging as the predominant driver of hepatic *vtg* transcription. Complementary to promoter-level regulation, recent work in the silver pomfret (*Pampus argenteus*) emphasized the importance of vitellogenin receptor (VGR) dynamics in shaping yolk accumulation. While *vtg* paralogs (*vtgAa*, *vtgAb*, *vtgC*) were primarily expressed in the liver, VGR expression in oocytes peaked during mid- to late vitellogenesis and localized to the plasma membrane, where it mediated VTG endocytosis [107]. This coordination between hepatic synthesis, plasma transport, and receptor availability in oocytes underscores that genomic diversification of *vtg* genes operates in tandem with the evolution of receptor-mediated uptake systems, ensuring efficient provisioning of yolk reserves during teleost reproduction.

### 3.3. Epigenetic Regulation of Sex-Specific Responses

Epigenetic mechanisms provide an additional layer of regulation, mediating both short- and long-term effects of EDCs. In *D. rerio*, the *vtg1* promoter is hypermethylated in males and hypomethylated in females under baseline conditions. EE2 exposure reduces promoter methylation in males, enabling robust induction of *vtg1* [108]. Genome-wide methylome analyses further show that EE2 alters methylation of steroidogenic loci, including *cyp19a1a*, *star*, and *hsd17b* [109]. Such changes can persist across generations. In *O. latipes*, ancestral exposure to BPA or EE2 led to reproductive defects in unexposed F2–F3 descendants, associated with stable germline methylation changes [110].

MicroRNAs also contribute. In *D. rerio* testes, EE2 activates the p53–miR-200 axis, altering the expression of genes involved in spermatogenesis and apoptosis, which in turn reduces sperm motility and compromises fertility [111]. Bisphenol S alters miRNAs targeting steroidogenic genes such as *cyp19a1a*, thereby interfering with estrogen signaling at the post-transcriptional level [112]. In *M. galloprovincialis*, BPA alters the expression of *dnmt* genes (DNA methyltransferases) [36], whereas in the oyster *Crassostrea gigas*, xenoestrogens disrupt non-coding RNAs and DNA methylation processes linked to gametogenesis [113]. Omics technologies expand this perspective by providing a system-level view of sex-specific responses. Transcriptomic studies in pipefish and salmonids show that EE2 upregulates female-biased genes such as *vtg*, *zp2*, and *zp3* (zona pellucida proteins), and *chg* (choriogenins), together with regulators of lipid metabolism. Males often display stronger transcriptional shifts, likely reflecting their lower baseline estrogen activity [114].

Proteomic analyses corroborate these findings: in male sheepshead minnow (*Cyprinodon variegatus*), EE2 induced hepatic remodeling including VTG isoforms and metabolic enzymes [115]; in European flounder (*P. flesus*), field proteomics revealed co-induction of VTG, choriogenins, and HSP70/90 in polluted estuaries [77]. Metabolomics adds further evidence: in *R. rutilus*, EE2 decreased circulating androgens and estrogens in males, impairing fertility [33,116]. In Atlantic cod (*G. morhua*), combined RNA-Seq and metabolomics revealed hepatic reprogramming affecting immunity and lipid metabolism [117].

Evidence from invertebrates indicates comparable trends. In *M. galloprovincialis*, BPA exposure alters early embryogenesis [36], induces VTG-like proteins in males (sometimes associated with oxidative stress rather than purely estrogenic signaling) [118], and reprograms female gonadal metabolism under environmentally relevant concentrations [119]. Despite the limited availability of integrated omics studies, combined proteomic and metabolomic analyses with other contaminants (e.g., BDE-47, TBBPA) consistently demonstrate disruptions in energy metabolism, osmoregulation (particularly in females), and stress pathways. Comparable approaches in bivalves exposed to metals corroborate these responses [120].

VTG induction should not be regarded as an isolated endpoint but rather as part of a broader molecular fingerprint of estrogenic exposure. Its interpretation requires integration with other molecular and physiological pathways affected by EDCs [38,75,121,122]. While most evidence currently derives from biomedical and toxicological models, extending multi-omics to aquatic species, particularly when combined with machine learning, holds promise for identifying conserved signatures of estrogenic exposure and for distinguishing sex- and species-specific responses. These approaches could substantially advance predictive ecotoxicology [121,122].

Collectively, the different omics layers provide complementary insights into sex-specific responses to EDCs. Transcriptomics highlights large-scale transcriptional remodeling, including sex-dependent regulation of *vtg* and steroidogenic genes; proteomics validates these patterns at the protein level and reveals post-translational modifications; metabolomics connects molecular changes with altered energy balance, lipid metabolism, and reproductive performance; and epigenomics uncovers persistent modifications, such as DNA methylation and microRNA regulation, that may underlie transgenerational effects. Importantly, these approaches are not independent: when combined in multi-omics frameworks, they enable the identification of co-regulated pathways, position VTG within broader molecular networks, and strengthen causal inference for regulatory ecotoxicology. An overview of representative transcriptomic, proteomic, metabolomic, and epigenomic endpoints is provided in a dedicated table (see Table 5, Section 5). Complementing this overview, Table 2 provides a synthesis of key genes and regulatory elements in fish, mollusks, and crustaceans, highlighting their functions, modes of regulation, and sex-dependent responses.

## 4. Vitellogenin as a Biomarker

VTG is one of the most established and sensitive biomarkers of endocrine disruption in aquatic species. Normally expressed in oviparous females under estrogenic control, its ectopic induction in males and juveniles provides a robust indicator of estrogen receptor activation. In this section, VTG is examined from molecular and genomic perspectives, considering its structure, regulation, induction mechanisms, sex-specific differences, ecotoxicological applications, and limitations.

### 4.1. Structure, Function, and Gene Regulation of VTG

VTG is synthesized in the liver under estrogenic control and is normally expressed only in oviparous females during vitellogenesis. Its induction in males and juveniles is a hallmark of exogenous estrogenic exposure, making VTG a central biomarker in both laboratory assays and field biomonitoring. VTGs are large glycolipophosphoproteins that supply amino acids, lipids, and minerals to developing embryos. After hepatic synthesis, they are secreted into the bloodstream, transported to the ovary, endocytosed by oocytes, and cleaved into yolk proteins (lipovitellin, phosvitin, β′-components), each with distinct nutritive and regulatory functions. Comparative genomics has revealed diversification of *vtg* genes following genome duplication events. In *D. rerio*, the Aa/Ab clades are strongly estrogen-inducible, whereas the C clade (*vtgC*) is only weakly responsive [101,123]. CRISPR–Cas9 knockout studies confirmed that Aa/Ab paralogs are essential for oocyte maturation and fertility, while *vtgC* plays a secondary role [102]. In salmonids, which underwent an additional whole-genome duplication, more than ten paralogs have been identified, often arranged in chromosomal clusters, with marked differences in estrogen responsiveness [124]. Promoter analyses have highlighted multiple estrogen response elements (EREs) bound primarily by ERα (*esr1*), while ERβ isoforms (*esr2a/b*) contribute to transcriptional modulation [106]. Epigenetic regulation further refines expression: in *D. rerio*, the *vtg1* promoter is hypermethylated in males and hypomethylated in females, and EE2 exposure reduces methylation in males, enabling strong induction [125]. Additional regulatory factors include HNF4α, a conserved hepatic transcription factor that regulates lipid and glucose metabolism and also contributes to the transcriptional control of *vtg* genes [126]. Among xenobiotic receptors, PXR is retained in several lineages and likely mediates crosstalk between VTG regulation, metabolism, and detoxification [127,128].

### 4.2. Mechanisms of Induction by EDCs and Receptor Crosstalk

Estrogens such as 17β-estradiol (E2) and synthetic analogs like EE2 activate ERα, which binds to *vtg* promoters and drives transcription. Robust VTG induction has been observed at environmentally relevant EE2 concentrations in *D. rerio*, *O. latipes*, and *P. promelas* [129,130]. Other xenoestrogens, including BPA and nonylphenol, also upregulate VTG, though with lower potency [131]. Crosstalk among signaling pathways modifies outcomes. Activation of AhR by dioxins or PCBs antagonizes ER signaling and suppresses VTG induction [97,132], whereas anti-androgens can enhance VTG expression [93]. Xenobiotic-sensing receptors, such as pregnane X receptor (PXR), reprogram estrogen-responsive networks and further modulate VTG regulation [119,120,121,122,123,124,125,126,127,128].

Comparable processes have been described in invertebrates. In *M. galloprovincialis*, nonylphenol exposure elevates VTG-like proteins in both sexes, commonly detected via alkali-labile phosphate assays [133]. BPA and related EDCs also induce VTG-like expression [15]. In crustaceans, organotins disrupt RXR-mediated pathways, interfering with metabolism and endocrine regulation [134]. RXR expression correlates with VTG during ovarian development, supporting its role in vitellogenesis [135].

### 4.3. Sex-Specific Differences in VTG Response

The diagnostic power of VTG stems from its sex-specific baseline. In females, VTG levels naturally rise during vitellogenesis and fluctuate with reproductive cycles, whereas in males and juveniles they remain negligible. This contrast makes contaminant-induced VTG expression a highly sensitive marker of estrogenic exposure. Laboratory studies consistently demonstrate stronger relative induction in males and juveniles, reflecting their low physiological background. Field observations corroborate this: in UK rivers impacted by wastewater effluents, male *R. rutilus* exhibited marked VTG elevation and intersex gonads, while females remained within physiological ranges. Juveniles also display heightened sensitivity, supporting their use as sentinel stages in ecotoxicological assays. Evidence from invertebrates indicates comparable trends, with VTG-like proteins detected in males following exposure to BPA, nonylphenol, or other xenoestrogens [19,34,136]. However, such responses can sometimes be confounded by generalized stress (e.g., oxidative stress), underscoring the need for careful interpretation. Overall, the sex-specific baseline of VTG remains one of its greatest strengths as a diagnostic endpoint, while variability in female physiology and potential non-estrogenic induction in invertebrates highlight its limitations.

### 4.4. Applications in Ecotoxicology

**Figure 1:**
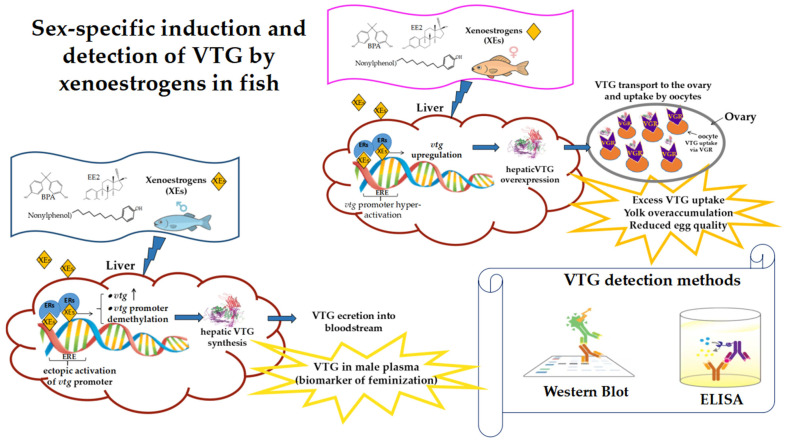
Schematic representation of sex-specific effects of xenoestrogens (XEs) on VTG expression in fish. Under normal conditions, males do not express VTG, whereas XE exposure induces *vtg* transcription and ectopic VTG synthesis. In females, where VTG is physiologically expressed, XEs enhance *vtg* upregulation and hepatic VTG synthesis. VTG is secreted into the bloodstream, transported to the ovary, and internalized by oocytes via vitellogenin receptors (VGRs). Examples of protein detection methods are shown (Western blot and ELISA); additional approaches are described in the text. Arrows indicate process direction; ↑ denotes *vtg* upregulation.

VTG is firmly established in ecotoxicological testing and regulatory frameworks, where it serves as a core endpoint in OECD Test Guidelines 229 (Fish Short-Term Reproduction Assay) and 234 (Fish Sexual Development Test) [86,87]. In these assays, VTG induction provides a sensitive readout of estrogenic activity and a mechanistic link between molecular signaling and reproductive outcomes. Several analytical methodologies are available. ELISA is the most widely applied method, providing high sensitivity and quantitative resolution, although it requires species-specific or cross-reactive antibodies that must be carefully validated. Western blotting offers semi-quantitative confirmation and is frequently used to verify ELISA results. At the transcript level, qPCR allows paralog-specific quantification of *vtg* genes, while RNA-Seq places VTG within broader co-expression networks, revealing links with genes involved in reproduction, metabolism, and stress responses. Non-invasive methods are emerging as innovative alternatives. Measurements of VTG in skin mucus or fin clips correlate strongly with plasma levels in several fish species, enabling more ethical, minimally invasive, and field-compatible monitoring strategies [136,137,138]. Biomonitoring studies consistently report elevated VTG levels and increased intersex prevalence in wild fish downstream of wastewater treatment plants, providing real-world evidence of estrogenic impacts. Within the AOP framework, ER activation constitutes the molecular initiating event, while *vtg* transcription and VTG protein synthesis represent early key events that connect molecular perturbation to impaired reproduction and altered population dynamics. VTG-like proteins are also being explored as biomarkers in bivalves and crustaceans, but their regulation remains poorly understood and their specificity as estrogenic indicators is debated [129,136]. Figure 1 integrates these methodological approaches with a schematic overview of the sex-specific induction of VTG by xenoestrogens in fish, highlighting detection through ELISA and Western blot. Despite its broad validation and regulatory use, the application of VTG as a biomarker is not without caveats. Interspecific variability, fluctuating baseline levels, and limitations in invertebrates highlight important challenges that need to be addressed, as discussed in the following section.

### 4.5. Limitations and Challenges

While VTG remains one of the most sensitive and widely validated biomarkers of estrogenic disruption, it is not suitable for evaluating the effects of several other classes of EDCs, including herbicides, anti-androgens, PFAS, organotins, and heavy metals, which act through distinct molecular pathways [122,139]. This underscores the importance of complementary biomarkers and multi-endpoint approaches to capture the full spectrum of endocrine disruption. Moreover, *vtg* is not expressed in the liver of most invertebrates, further limiting its use as a cross-phyla biomarker and highlighting the need for taxa-specific endpoints [19,37].

VTG is a robust and widely validated biomarker, but its application is not without challenges. Interspecific variation in *vtg* gene repertoires and differential estrogen-responsiveness of paralogs result in divergent inducibility profiles, complicating cross-species comparisons. In females, baseline fluctuations during reproductive cycles may obscure contaminant-induced responses, making interpretation highly context-dependent. Moreover, thresholds for ecological risk remain poorly defined: moderate VTG induction does not always predict reproductive impairment, indicating that induction levels cannot be linearly extrapolated to reproductive outcomes. Environmental mixtures add further complexity. AhR activation can antagonize ER-mediated VTG expression, whereas anti-androgens may potentiate it, and the net effect depends on interactions across multiple signaling pathways. Technical issues also persist, including antibody specificity, lack of standardized reference materials, integration of omics data, and inter-laboratory variability. Epigenetic regulation introduces an additional layer of complexity, since EE2 reduces *vtg1* promoter methylation in *D. rerio*, while BPA alters DNA methylation and *dnmt* expression in *M. galloprovincialis*. These experimental findings illustrate how EDCs directly interfere with epigenetic machinery. More broadly, recent reviews emphasize that such molecular and epigenetic perturbations must be interpreted within wider mechanistic and ecological context [40,136].

### 4.6. Evolutionary and Biotechnological Perspectives of VTG

The diversification of *vtg* paralogs in teleosts and the occurrence of VTG-like proteins in mollusks and crustaceans reflect a conserved evolutionary strategy for yolk precursor synthesis under endocrine control. This variability underscores the ecogenomic significance of VTG: species with highly inducible paralogs act as sensitive sentinels of estrogenic pollution, whereas species with weakly inducible *vtg* genes may underestimate ecological risk. Beyond its ecological relevance, VTG also holds biotechnological potential. Advances in antibody development and multiplex immunoassays have improved diagnostic precision, while *vtg* promoters have been incorporated into biosensors and transgenic *D. rerio* reporters for real-time detection of estrogenic activity. Integrating these molecular tools with omics platforms and predictive models, including machine learning, could enhance assay sensitivity, enable species-specific customization, and expand monitoring capacities. Altogether, VTG remains a pivotal biomarker of endocrine disruption, but its greatest value emerges when interpreted in combination with complementary endpoints within multi-biomarker frameworks [79,107,138]. Table 3 summarizes *vtg* genes and paralogs across taxa, highlighting their physiological induction in females, induction under EDC exposure in males and juveniles, and the main regulatory pathways involved. The information is based on the studies presented in Section 4.

## 5. Integration with Other Biomarkers and Molecular Endpoints

Although VTG is a highly sensitive biomarker of estrogenic disruption, it reflects only part of the broader molecular and physiological networks affected by EDCs. Because real-world exposures typically involve mixtures acting through multiple pathways, integrating VTG with complementary endpoints enhances mechanistic specificity and ecological relevance.

### 5.1. Co-Expression with Hormone Metabolism and Stress Genes

In fish, VTG induction frequently coincides with the up-regulation of female-biased reproductive genes such as *zp* and *chg*, together with estrogen-responsive hepatic metabolic programs [140,141]. Conversely, activation of cyp1a by AhR agonists (e.g., dioxin-like PCBs) can antagonize ER-driven VTG responses, underscoring the importance of mixture effects and receptor cross-talk [98,140]. Stress proteins (HSP70/90) are also commonly detected alongside VTG in experimental and field studies. While non-specific, they strengthen WoE panels when interpreted in exposure context. For example, in mullet (*Mugil cephalus*), hepatic HSP70 and CYP1A reliably tracked contaminant exposure across estuaries [70,142].

### 5.2. Epigenetic and Transcriptomic Markers

Epigenetic endpoints extend interpretation beyond acute transcriptional changes. In adult male *D. rerio*, EE2 reduces CpG methylation at the *vtg1* promoter, establishing a persistent hypomethylated state that facilitates robust re-induction upon re-exposure [143]. Across taxa, EDCs modulate DNA methylation machinery (e.g., *dnmt* genes) and non-coding RNAs (ncRNAs) involved in steroidogenesis and gametogenesis. Transcriptomic studies in flatfish and cyprinids consistently show that *vtg* induction co-occurs with *zp* and *chg* genes, together with immune and metabolic pathways, defining a broader “estrogenic signature” rather than an isolated endpoint [140,141]. In bivalves, BPA perturbs early embryonic transcription and produces sex-dependent metabolic shifts in gonads, reinforcing the value of combining VTG-like signals with multi-omics data [19,119].

### 5.3. Multi-Biomarker and AOP Approaches

Embedding VTG within WoE frameworks that integrate histological endpoints (e.g., intersex), receptor- and detoxification-pathway markers (e.g., cyp1a/CYP1A, HSPs), and omics data strengthens causal inference under complex exposure scenarios. Field proteomics in caged *P. flesus* revealed coordinated hepatic protein shifts consistent with mixed-contaminant stress, thereby complementing VTG-based diagnostics [144].

Within the AOP framework, ER activation constitutes the molecular initiating event (MIE), whereas increased *vtg* transcription and VTG protein synthesis represent early key events (KEs) that contribute to downstream gonadal pathology and reduced reproductive performance at organism and, ultimately, population levels [29,145].

Across taxa, EDCs also affect invertebrate biomarkers. In mollusks, alterations in DNA methyltransferases (DNMTs) and non-coding RNAs (ncRNAs) have been linked to disrupted gametogenesis [19,37], while in crustaceans, endocrine disruptors interfere with the EcR/RXR pathway and the methyl farnesoate (MF)–20-hydroxyecdysone (20E) axis, impairing molting and reproduction [83,84]. Table 4 summarizes key sex-specific biomarkers associated with EDCs in aquatic organisms, including their responses in males and females, detection methods, and ecotoxicological relevance.

Given the complexity of mixture exposures, integrative frameworks are increasingly required; the advantages and limitations of different omics strategies are outlined in Table 5.

## 6. Ecotoxicological and Ecogenomic Implications for Risk Assessment

The integration of molecular biomarkers such as VTG with complementary endpoints provides mechanistic insight into endocrine disruption, but a major challenge remains translating these signals into ecologically meaningful outcomes. In aquatic ecosystems, where organisms are chronically exposed to complex contaminant mixtures, risk assessment requires frameworks that link molecular events to adverse outcomes at population and community levels.

### 6.1. Species-Specific Sensitivity in Marine Fish

Marine fish display pronounced interspecific variability in their responses to endocrine disruptors. *P. flesus* consistently exhibits estrogenic fingerprints in impacted estuaries, with induction of VTG, choriogenins, and zona pellucida proteins in males, often accompanied by intersex development [150,151]. In *M. cephalus*, exposure to contaminated estuaries triggers broad transcriptional remodeling that integrates estrogenic, stress-related, and metabolic pathways, with co-induction of VTG, CYP1A, and HSP70 [70,152]. Recent studies also show that *M. cephalus* hepatocytes exposed to perfluorononanoic acid (PFNA) undergo estrogenic activation, with induction of *vtg* and estrogen receptor transcripts [153]. Seasonal and contaminant-driven shifts in liver and gonadal lipids further highlight metabolic involvement [154]. Field surveys confirm up-regulation of *cyp19a1a* and VTG, together with intersex in male *M. cephalus*, reinforcing their value as sentinel species [155]. In sea bass (*Dicentrarchus labrax*), responses are sex-specific: males exhibit robust VTG induction and epigenetic modifications, whereas females display subtler transcriptional shifts [156]. In *G. morhua*, EE2 exposure induces systemic hepatic reprogramming, altering immune and metabolic pathways with direct consequences for reproduction and health [157]. Collectively, these examples underline the importance of considering species-specific genomic and epigenomic repertoires in monitoring strategies.

### 6.2. Reproductive Fitness and Population-Level Effects

At the organismal level, molecular perturbations scale up to reproductive impairment. Long-term surveys of *R. rutilus* in UK rivers demonstrated clear links between feminization, intersex, impaired fertility, and exposure to estrogenic effluents [148]. A landmark whole-lake experiment in Canada revealed that chronic EE2 exposure caused *P. promelas* population collapse, with recovery only after removal of the contaminant source [149]. Comparable findings in *P. flesus*, *M. cephalus*, and *G. morhua* confirm that endocrine disruption impairs fecundity, sperm quality, oogenesis, and fertilization success, thereby bridging molecular biomarkers with demographic consequences [150,154,157].

### 6.3. Implications for Biodiversity and Ecosystem Health

The consequences of endocrine disruption extend beyond individuals to populations and ecosystems. Impaired reproduction in *M. cephalus* disrupts nutrient cycling and trophic transfer, while effects on commercially important species such as *G. morhua* and *D. labrax* raise concerns for fisheries sustainability.

In bivalves and oysters, altered vitellogenesis and gametogenesis compromise benthic–pelagic coupling and biofiltration. In crustaceans, interference with ecdysteroid-regulated molting and reproduction emphasizes ecosystem-level vulnerability [5,61]. While research on endocrine disruption in fish is extensive and has provided standardized biomarkers such as VTG, studies in mollusks and crustaceans are comparatively scarce and fragmented. This discrepancy highlights the urgent need for further investigations in non-fish taxa to broaden ecotoxicological paradigms. These findings underscore the importance of ecogenomic perspectives in predicting community shifts and biodiversity loss under contaminant pressure [8,158].

### 6.4. Integrating Molecular Data into Risk Assessment

Risk assessment frameworks are increasingly embedding molecular and organismal endpoints into regulatory contexts. Within the AOP framework, VTG induction is positioned as a key event linking ER activation to reproductive impairment and eventual population decline [159]. Advances in quantitative AOPs and networked AOPs are enhancing predictive capacity, whereas integrative strategies that combine biomarkers, histology, and chemical analyses strengthen causal inference.

## 7. Future Perspectives

Research on endocrine disruption in aquatic organisms has progressed from reliance on single biomarkers such as VTG toward integrated, multi-layered strategies that capture the complexity of sex- and species-specific responses. Nevertheless, significant knowledge gaps remain in developing diagnostic tools tailored to sex and developmental stage, exploiting multi-omics and big-data resources, and incorporating evolutionary and ecological dimensions. At the same time, embedding molecular endpoints into regulatory frameworks and sustainability agendas represents both a challenge and an opportunity.

### 7.1. Development of Sex-Specific Molecular Biomarkers

Although VTG induction in males and juveniles remains among the most sensitive indicators of estrogenic exposure, interpretation in females is complicated by natural reproductive cycles and high baseline levels. Future work should therefore prioritize sex-specific biomarker panels. Promising candidates include ER isoforms (*esr1*, *esr2a*, *esr2b*), oocyte-maturation genes (*zp2*, *zp3*), spermatogenesis markers (*spag*, *sycp3*), and sex-biased miRNAs regulating steroidogenesis and gametogenesis. Epigenetic signatures, such as sex-specific methylation patterns in *vtg1* promoters or in steroidogenic regulators (*cyp19a1a*, *hsd17b*, *star*), also represent relatively stable biomarkers [147,160].

Establishing panels that discriminate male, female, and juvenile responses will increase diagnostic precision and reveal vulnerabilities masked when only VTG is measured. VTG will remain a cornerstone biomarker, but its diagnostic value can be enhanced by integrating omics signatures and improving immunoassay sensitivity and specificity. Recent advances include the development of species-specific assays in *D. rerio* and cyprinids [129,146], and customized antibodies against vitellogenin mRNA in crustaceans such as *Palinurus elephas* [161]. These strategies strengthen VTG-based diagnostics and support its central role in predictive and regulatory ecotoxicology.

### 7.2. Applications of Multi-Omics and Big Data

Multi-omics technologies now enable system-level profiling of endocrine disruption. Transcriptomic analyses in fish exposed to EE2 revealed genome-wide estrogenic signatures [114], while integrated transcriptomic–proteomic studies in *D. rerio* uncovered concurrent estrogenic and metabolic shifts [162]. In *S. taeniatus*, combined transcriptomic–metabolomic approaches identified key regulators of vitellogenesis [163]. Reviews highlight the growing use of triple-omics workflows to capture cross-taxa fingerprints [158], and proteomics of fish scales has identified estrogen-responsive markers for non-invasive diagnostics [164]. Transcriptomic approaches have identified toxicogenomic signatures, defined as conserved patterns of gene expression associated with specific contaminant classes or modes of action. These signatures further demonstrate the potential of omics integration to provide mechanistic insights and to distinguish between different exposure scenarios [165]. The next step is to translate these complex datasets into predictive tools. Machine learning and network analysis can integrate transcriptomic, proteomic, metabolomic, and epigenomic data to identify exposure signatures that are conserved yet sex-specific. Comparative ecogenomic databases will support cross-species predictions and help distinguish predominantly estrogenic exposures from mixed-contaminant profiles. These strategies will move ecotoxicology from descriptive diagnostics toward models capable of forecasting population-level outcomes.

Recent advances illustrate this potential. High-throughput transcriptomics (HTTr) in larval *P. promelas* generated transcriptomic points-of-departure (tPODs) that were comparable to—or more sensitive than—apical endpoints, facilitating rapid screening of contaminants such as PFAS [165,166,167,168]. Integrated multi-omics pipelines combined with machine learning are also being refined to improve robustness, interpretability, and regulatory acceptance [169,170,171].

### 7.3. Ecogenomics and Adaptive Evolution

An emerging focus concerns the evolutionary consequences of chronic exposure to EDCs. Variation in *vtg* paralogs, ER isoforms, and steroidogenic pathways across teleosts reflects both historical genome duplication and local adaptation. Increasing evidence suggests that populations chronically exposed to EDCs can undergo evolutionary shifts. Early studies mainly documented biochemical and epigenetic changes, such as altered steroidogenesis, promoter methylation, and microRNA regulation, which may persist across generations. In addition, population-level studies have demonstrated genetic adaptation to contaminant exposure. For example, killifish (*Fundulus heteroclitus*) populations inhabiting polluted estuaries have evolved resistance to dioxin-like compounds through adaptive variants in the aryl hydrocarbon receptor (AHR) pathway [172]. More broadly, evolutionary toxicology research has documented allelic shifts and genetic divergence in aquatic organisms chronically exposed to toxicants, confirming that long-term contaminant exposure can drive heritable evolutionary changes [173].

For example, *R. rutilus* populations in estrogen-contaminated UK rivers show altered baseline expression of *vtg* and ER genes [161,162], while *M. galloprovincialis* in polluted harbors display modified methylation profiles consistent with long-term selection [133]. Such adjustments may enhance survival under chronic stress but at the cost of reduced reproductive plasticity and resilience.

Future studies should employ genome-wide association studies (GWAS), population transcriptomics, and epigenetic-landscape analyses to clarify how adaptive responses shape biodiversity and persistence. Recent work also highlights the potential of DNA-methylation markers combined with machine learning as predictors of sex and stress history in fish [156,165,174]. Ecogenomics thus bridges molecular biomarkers and evolutionary ecology, showing how pollution not only impairs reproduction but can also drive long-term genetic and epigenetic change.

The stronger focus on fish compared to invertebrates reflects the current state of the literature, as molecular and omics studies in mollusks and crustaceans remain limited. Expanding research on these taxa is essential for a more comprehensive ecotoxicological perspective.

### 7.4. Regulatory and Sustainability Perspectives

Integrating molecular biomarkers into regulatory frameworks is increasingly recognized as a priority. The AOP framework formalizes links between MIE and population-level effects, and advances in quantitative and networked AOPs are strengthening predictive toxicology [159,175]. WoE approaches further enhance causal inference by combining molecular endpoints with histopathology and ecological data [176].

Future guidelines should incorporate omics-based signatures, epigenetic endpoints, and sex-specific panels to enhance sensitivity and predictive value. International initiatives, such as OECD recommendations, the EU Water Framework Directive, the Marine Strategy Framework Directive, and the US EPA’s EDSP program, already indicate regulatory uptake of molecular and genomic data [169,177,178]. Importantly, these efforts align with the United Nations SDG 14, underscoring how molecular ecotoxicology can provide both mechanistic insight and actionable strategies for biodiversity conservation and ecosystem management.

## 8. Conclusions

EDCs remain a critical environmental threat due to their ability to disrupt hormone-regulated pathways central to reproduction, development, and population stability in aquatic organisms. Among biomarkers of estrogenic disruption, VTG is the most sensitive and widely validated, providing a direct mechanistic link between estrogen receptor activation and reproductive impairment. Its diagnostic and predictive value is maximized when integrated into broader molecular, genomic, and physiological networks, and supported by epigenetic and multi-omics evidence. The ecogenomic perspective further demonstrates how differences in genomic architecture and regulatory repertoires shape species-specific sensitivity, with long-term studies such as those on *R. rutilus* in UK rivers illustrating the ecological benefits of reducing estrogenic effluents.

Looking forward, priorities include developing sex-specific biomarker panels, integrating multi-omics with machine learning, and expanding research on invertebrates to capture cross-phyla responses. Such integration will enhance ecological risk assessment and provide more robust tools for regulatory decision-making. Equally important is embedding molecular endpoints into regulatory frameworks, harmonizing protocols across regions, and aligning with international sustainability goals such as SDG 14.

In conclusion, VTG exemplifies how molecular biomarkers can transform ecotoxicology from a descriptive to a predictive science, providing actionable strategies for biodiversity conservation, ecosystem sustainability, and evidence-based environmental governance.

## Figures and Tables

**Table 1:** Representative examples of endocrine disruption in aquatic taxa. The table includes freshwater and marine fish, mollusks, and crustaceans, summarizing key EDCs, their biological effects, and sex-specific responses.

Taxonomic Group	EDCs	Main Effects	Sex-Specific Effects	Representative References
Fish (*P. promelas*)	17α-ethinylestradiol (EE2)	VTG induction, intersex development, population collapse in whole-lake experiment	Males strongly induced; females affected at reproductive stages	[38,39]
Fish (*R. rutilus*)	Nonylphenol, wastewater effluents	VTG induction, intersex gonads, feminization of wild populations	Feminization in males; females within physiological baseline	[34,35]
Fish (various freshwater species)	Atrazine	Aromatase induction, altered sex steroid balance, feminization	Males overexpress aromatase; feminization observed	[32,33]
Fish *(G. morhua*)	17α-ethinylestradiol (EE2)	Hepatic reprogramming of lipid metabolism and immune pathways (multi-omics)	Sex-biased transcriptional shifts, stronger in males	[76]
Fish (*P. flesus*)	Field exposure in polluted estuaries	Co-induction of VTG, choriogenins, HSP70/90; evidence of estrogenic disruption	Male induction of VTG/Chg; females less responsive	[77]
Mollusks (*Mytilus* spp.)	Bisphenol A (BPA), EE2	Disrupted embryogenesis, altered lipid metabolism and vitellogenesis; inconsistent VTG-like induction	Sex-dependent responses unclear; variable results among studies	[36,37,40,41,71]
Mollusks (*Crassostrea gigas*)	Xenoestrogens (e.g., BPA, EE2)	Altered DNA methylation and non-coding RNAs; disruption of gametogenesis	Epigenetic changes may affect gametes differently between sexes	[45]
Crustaceans (general, e.g., crabs, amphipods)	Organotins, pesticides	Disruption of EcR/RXR signaling; impaired molting, vitellogenesis, and reproduction	Sex-dependent reproductive impairment observed	[79,80,81,82,83]
Crustaceans (*Marsupenaeus japonicus*)	Methyl farnesoate (MF), 20-hydroxyecdysone (20E)	Experimental exposure confirmed disruption of molting and reproduction (EcR/RXR axis)	Disruption of hormone balance affects both sexes differently	[84]

**Table 2:** Key genes and regulatory elements affected by EDCs in fish and aquatic invertebrates, with associated functions, mechanisms, and sex-specific responses where known. Arrows (→) indicate the direction of molecular or physiological changes.

Gene	Gene Product and Function	Main EDCs	Regulation by EDCs	Molecular Mechanism	Sex-Specific Responses	References
*vtg* (*D. rerio*, salmonids, *R. rutilus*)	VTG (yolk precursor protein)	EE2, BPA, NP	Strongly upregulated	ERα → EREs; promoter hypomethylation enhances induction	Negligible baseline in males → strong induction under EDCs; physiological in females	[100,101,102,103,104,105,106,107]
*zp2*, *zp3* (teleosts)	ZP2/ZP3 (zona pellucida proteins, egg envelope)	EE2, BPA	Upregulated	ER-dependent transcription; co-regulated with *vtg*	Female-biased; weak/no expression in males under control conditions	[88,90]
*chg* (teleosts)	Chg (choriogenins, egg envelope glycoproteins)	EE2, NP	Upregulated	ERα-mediated transcription	Strong biomarker in males (ectopic induction)	[106,107]
*cyp19a1a* (teleosts, amphibians)	CYP19A1A (Aromatase A, estrogen synthesis)	Atrazine, BPA	Induced	Steroidogenesis; conversion androgens → estrogens	Overexpression in males → feminization	[91,110]
*cyp1a* (teleosts, invertebrates)	CYP1A (cytochrome P450 1A, detoxification enzyme)	Dioxins, PCBs	Upregulated	AhR/ARNT → XREs; antagonizes ER signaling	Suppression of *vtg* in males	[97,98]
*star* (teleosts)	StAR (steroidogenic acute regulatory protein, cholesterol transport)	BPA, metals	Altered	Steroid hormone biosynthesis	Affects sex steroid balance in both sexes	[109]
*hsd* family (teleosts)	HSDs (hydroxysteroid dehydrogenases, steroid metabolism)	BPA, metals	Modulated	Steroidogenesis	Key in androgen/estrogen ratio; dysregulation impacts fertility	[91,110]
*dnmt* (teleosts, mollusks)	DNMTs (DNA methyltransferases, epigenetic regulators)	BPA, EE2	Altered	DNA methylation changes at promoters (*vtg1*, *cyp19a1a*)	Sex-specific promoter methylation (e.g., *vtg1* hypo in females, hyper in males)	[108,113]
miR-200 family (*D. rerio*)	miR-200 family (epigenetic regulators of spermatogenesis)	EE2	Upregulated	p53–miR-200 axis	Reduced sperm motility in males	[111,112]
*vtg*-like (bivalves, e.g., *Mytilus* spp.)	VTG-like yolk precursor proteins	BPA, EE2	Inconsistent induction	Estrogenic signaling vs. oxidative stress cross-talk	Variable; induction sometimes absent in males/females	[118,119,120]
EcR/RXR (crustaceans)	Ecdysone receptor/Retinoid X receptor (molting, reproduction)	Organotins, pesticides	Disrupted	Interference with 20E–MF axis	Impaired molting and vitellogenesis; sex-dependent reproductive impairment	[92,93,121,122]

**Table 3:** VTG genes and paralogs in different taxa, their physiological induction in females, induction under EDCs in males and juveniles, main regulatory pathways, and relevant notes.

VTG Genes and Paralogs (Species)	Physiological Induction (Females)	Induction Under EDC Exposure (Males and Juveniles)	Main Regulatory Pathway	Notes	References
*vtgAa*, *vtgAb* (*D. rerio*)	Strong induction during vitellogenesis under estrogen control	Robust induction by EE2, BPA, Nonylphenol	ERα binding EREs; promoter demethylation	Essential for oocyte maturation and fertility	[100,102,123,125,129,130,137]
*vtgC* (*D. rerio*)	Weakly responsive in females	Minimal induction under EDCs	Weak ERα responsiveness	Secondary role; subfunctionalized paralog	[101,104,105]
*multiple vtg paralogs (salmonids*, e.g., *Salmo salar*)	Several paralogs highly induced; others weak	Differential induction depending on paralog	ERα/EREs; subfunctionalization	Large gene arrays due to genome duplication	[103,105,106]
VTG-like proteins (*Mytilus* spp.)	Baseline expression variable	Sometimes induced by BPA, Nonylphenol, EE2 (inconsistent)	ER-related transcripts; stress pathways	Biomarker value debated, not consistent	[118,119,120,133]
VTG-like transcripts (crustaceans, e.g., crabs, shrimps)	VTG expression increases during female vitellogenesis under hormonal control	Limited induction after organotin and other EDC exposure; responses vary across taxa	RXR/EcR pathways; interaction with methyl farnesoate and 20-hydroxyecdysone	VTG investigated as a potential biomarker in crustaceans, but responses remain variable across taxa	[21,84,85,135]

**Table 4:** Sex-specific biomarkers associated with endocrine disruption in aquatic organisms. The table integrates evidence from fish, mollusks, and crustaceans, summarizing biomarker responses in males and females, detection methods, and their ecotoxicological relevance.

Biomarker	Species and Taxa	Response in Males	Response in Females	Assay	Ecotoxicological Relevance	References
VTG	Fish (Cyprinis, Salmonids)	Strong induction	Baseline fluctuation	ELISA, qPCR, Western blot	Sensitive biomarker of estrogenic exposure	[15,16,17,18,106,136,137,146]
ERα/ERβ isoforms	Teleosts	Differential modulation	Variable (cycle-dependent)	qPCR, RNA-Seq	Key regulators of VTG and reproduction	[88,89,90,125]
miR-200 cluster	*D. rerio*	Reduced sperm motility	Not determined	qPCR, small RNA-seq	Epigenetic marker of fertility disruption	[111,112,147]
DNMTs/ncRNAs	Mollusks (*Mytilus*, *Crassostrea*)	Altered methylation, disrupted ncRNAs	Gametogenesis impairment	qPCR, methylome analysis	Epigenetic biomarkers of endocrine disruption in invertebrates	[20,36,37,113]
EcR/RXR signaling	Crustaceans (crabs, amphipods, shrimps)	Impaired molting and reproduction	Altered vitellogenesis	Transcriptomics, receptor assays	Biomarkers of endocrine disruption via the MF/20E hormonal pathway	[21,60,84,85,135]

**Table 5:** Omics approaches in aquatic ecotoxicology of endocrine disruptors. The table summarizes transcriptomic, proteomic, metabolomic, and epigenomic strategies, highlighting their main advantages, limitations, representative case studies, and relevance to sex-specific responses and VTG regulation.

Omics Approach	Advantages	Limitations	Representative Examples	Relevance to Sex-Specific Responses and VTG	References
Transcriptomics	Genome-wide sensitivity; reveals estrogenic signatures; identifies co-regulated pathways	High data complexity; requires bioinformatic pipelines	EE2—*D. rerio* (estrogenic signatures); RNA-Seq—*G. morhua* (immune/metabolic reprogramming); *M. edulis* (biomarkers of endocrine disruption)	Sex-biased regulation of *vtg*, *zp2/3*, *chg*; stronger shifts in males due to lower baseline estrogen	[24,117,148,149]
Proteomics	Links directly to protein biomarkers; detects post-translational modifications	Requires species-specific antibodies; less standardized across taxa	Male *C. variegatus*—hepatic remodeling under EE2; *P. flesus* (field proteomics: VTG, Chg, HSP70/90); *M. galloprovincialis* (oxidative stress)	Confirms VTG isoform induction; links sex-specific stress to protein remodeling	[11,12,120,140,141]
Metabolomics	Captures functional metabolic shifts; sensitive to physiological disruption	Lower mechanistic specificity; metabolites can be transient	*R. rutilus*—EE2 reduced circulating steroids; *G. morhua* (multi-omics: systemic reprogramming); mussels (BDE-47/TBBPA: altered energy metabolism)	Sex-dependent metabolic trade-offs; VTG synthesis linked to lipid/energy metabolism	[24,25,119]
Epigenomics	Reveals persistent and transgenerational effects; potential biomarkers	Limited cross-species data; methods still emerging in ecotox	*D. rerio*—*vtg1* promoter methylation under EE2; *O. latipes*—methylation shifts (BPA/EE2); *M. galloprovincialis*—altered *dnmt*; *C. gigas*—ncRNA disruption	Explains sex-specific sensitivity (promoter methylation differences); reveals heritable effects beyond VTG induction	[10,73,111,112,143]

## Data Availability

No new data were created or analyzed in this study. Data sharing is not applicable to this article.

## Abbreviations

The following abbreviations are used in this manuscript:

ABC	ATP-Binding Cassette (transporters)
AOP	Adverse Outcome Pathway
AOPs	Adverse Outcome Pathways
AR	Androgen Receptor
ARα	Androgen Receptor alpha
ARβ	Androgen Receptor beta
ATP	Adenosine Triphosphate
AhR	Aryl Hydrocarbon Receptor
BDE	Brominated Diphenyl Ether
BPA	Bisphenol A
CRISPR	Clustered Regularly Interspaced Short Palindromic Repeats
CYP1A	Cytochrome P450 Family 1 Subfamily A
Chg	Choriogenins
CpG	Cytosine-phosphate-Guanine dinucleotide
DDT	Dichlorodiphenyltrichloroethane
DNA	Deoxyribonucleic Acid
E2	17β-Estradiol
EDC	Endocrine-Disrupting Chemical
EDCs	Endocrine-Disrupting Chemicals
EDSP	Endocrine Disruptor Screening Program (US EPA)
EE2	17α-Ethinylestradiol
ELISA	Enzyme-Linked Immunosorbent Assay
ER	Estrogen Receptor
ERE	Estrogen Response Element
ERα	Estrogen Receptor alpha
ERβ	Estrogen Receptor beta
esr2a	Estrogen Receptor beta a (teleost isoform)
esr2b	Estrogen Receptor beta b (teleost isoform)
EU	European Union
GPER	G Protein-Coupled Estrogen Receptor
GWAS	Genome-Wide Association Study
HNF4α	Hepatocyte Nuclear Factor 4 alpha
HSP70	Heat Shock Protein 70
HSP90	Heat Shock Protein 90
HSPs	Heat Shock Proteins
HTTr	High-Throughput Transcriptomics
MIE	Molecular Initiating Event (AOP framework)
OECD	Organisation for Economic Co-operation and Development
PCB	Polychlorinated Biphenyl
PFAS	Per- and Polyfluoroalkyl Substances
PFNA	Perfluorononanoic Acid
PXR	Pregnane X Receptor
RNA	Ribonucleic Acid
RNA-Seq	RNA Sequencing
RXR	Retinoid X Receptor
SDG	Sustainable Development Goal
SLC	Solute Carrier Transporter
StAR	Steroidogenic Acute Regulatory Protein
tPOD	Transcriptomic Point of Departure
VTG	Vitellogenin
WoE	Weight of Evidence
ZP2	Zona Pellucida Glycoprotein 2
ZP3	Zona Pellucida Glycoprotein 3
cyp19a1a	Cytochrome P450 Family 19 Subfamily A Member 1a (ovarian aromatase)
cyp19a1b	Cytochrome P450 Family 19 Subfamily A Member 1b (brain aromatase)
dnmt	DNA Methyltransferase
mPRα	Membrane Progestin Receptor alpha
miR-200	MicroRNA-200 family
ncRNA	Non-Coding RNA
qPCR	Quantitative Polymerase Chain Reaction
